# Transcriptomics analysis revealed an indirect effect of aqueous cigarette smoke extract in promoting the adhesion of monocytic cells to endothelial cells

**DOI:** 10.1186/1753-6561-6-S6-P32

**Published:** 2012-10-01

**Authors:** Carine Poussin, Inka Gallitz, Walter Schlage, Yvonne Steffen, Katrin Stolle, Stephan Lebrun, Julia Hoeng, Michael Lietz

**Affiliations:** 1 Philip Morris International R&D, Philip Morris Products S.A., Quai Jeanrenaud 5, 2000 Neuchâtel, Switzerland; 2 Philip Morris International R&D, Philip Morris Research Laboratories GmbH, Fuggerstrasse 3, 51149 Cologne, Germany

## Background

The adhesion of monocytic cells to the 'dysfunctional' endothelium constitutes a critical step in the initiation of atherosclerosis.

Cigarette smoke (CS) has been shown to contribute to the monocyte-endothelial adhesion process. However, the complex underlying molecular mechanisms remain to be unraveled.

## Materials and methods

To investigate the impact of CS on the adhesion of monocytic cells to the endothelium, we developed a conditioned-medium experiment combined with an *in vitro* adhesion assay intended to mimic the situation found in the systemic compartment. Using a transcriptomics approach followed by confirmation experiments, we were able to identify a key mechanism by which aqueous CS extract in the form of smoke-bubbled phosphate buffered saline (sbPBS) promotes the adhesion of monocytic mono mac 6 (MM6) cells to human umbilical vein endothelial cells (HUVECs).

## Results

While soluble CS constituents elicit a strong oxidative stress response in both cell types, the induced expression of E-selectin, vascular cell adhesion molecule-1 (VCAM-1)and intercellular adhesion molecule-1 (ICAM-1) responsible for the binding of MM6 cells to HUVECs occurs through a pro-inflammatory paracrine effect. Our results show that this effect is largely driven by tumor necrosis factor α (TNF α) produced by MM6 cells exposed to sbPBS.

## Conclusions

Our findings demonstrate that the adhesion of monocytic cells to endothelial cells is promoted through an indirect effect of sbPBS, mainly involving a key soluble factor TNF α and open new avenues for translational research in the comprehension of atherosclerosis development.

